# Supplementary material for: Characterising within-hospital SARS-CoV-2 transmission events using epidemiological and viral genomic data across two pandemic waves
Source: Nat Commun. 2022 Feb 3;13:671. doi: 10.1038/s41467-022-28291-y (PMC8814040; doi:10.1038/s41467-022-28291-y)
Supplement: Supplementary file 3 — Description of Additional Supplementary Files [file 41467_2022_28291_MOESM3_ESM.pdf]

Description of Additional Supplementary Files

File Name: Supplementary Data 1
Description: List of European Nucleotide Archive (ENA) accession numbers for all SARS-CoV-2 sequence data used in the study
